# Time and frequency dependent changes in resting state EEG functional connectivity following lipopolysaccharide challenge in rats

**DOI:** 10.1371/journal.pone.0206985

**Published:** 2018-11-12

**Authors:** Matthew A. Albrecht, Chloe N. Vaughn, Molly A. Erickson, Sarah M. Clark, Leonardo H. Tonelli

**Affiliations:** 1 Laboratory of Behavioral Neuroimmunology, Department of Psychiatry, University of Maryland School of Medicine, Baltimore, MD, United States of America; 2 School of Public Health, Curtin Health Innovation Research Institute (CHIRI), Curtin University, Perth, Western Australia, Australia; 3 Maryland Psychiatric Research Center, Department of Psychiatry, University of Maryland School of Medicine, Baltimore, MD, United States of America; University Technology Petronas, MALAYSIA

## Abstract

Research has shown that inflammatory processes affect brain function and behavior through several neuroimmune pathways. However, high order brain functions affected by inflammation largely remain to be defined. Resting state functional connectivity of synchronized oscillatory activity is a valid approach to understand network processing and high order brain function under different experimental conditions. In the present study multi-electrode EEG recording in awake, freely moving rats was used to study resting state connectivity after administration of lipopolysaccharides (LPS). Male Wistar rats were implanted with 10 cortical surface electrodes and administered with LPS (2 mg/kg) and monitored for symptoms of sickness at 3, 6 and 24 h. Resting state connectivity and power were computed at baseline, 6 and 24 h. Three prominent connectivity bands were identified using a method resistant to spurious correlation: alpha (5–15 Hz), beta-gamma (20–80 Hz), and high frequency oscillation (150–200 Hz). The most prominent connectivity band, alpha, was strongly reduced 6 h after LPS administration, and returned to baseline at 24 h. Beta-gamma connectivity was also reduced at 6 h and remained reduced at 24 h. Interestingly, high frequency oscillation connectivity remained unchanged at 6 h and was impaired 24 h after LPS challenge. Expected elevations in delta and theta power were observed at 6 h after LPS administration, when behavioral symptoms of sickness were maximal. Notably, gamma and high frequency power were reduced 6 h after LPS and returned to baseline by 24 h, when the effects on connectivity were more evident. Finally, increases in cross-frequency coupling elicited by LPS were detected at 6 h for theta-gamma and at 24 h for theta-high frequency oscillations. These studies show that LPS challenge profoundly affects EEG connectivity across all identified bands in a time-dependent manner indicating that inflammatory processes disrupt both bottom-up and top-down communication across the cortex during the peak and resolution of inflammation.

## Introduction

Extensive research has shown that inflammatory processes affect brain function and behavior via several neuroimmune mechanisms that integrate the central nervous (CNS) and immune systems in response to environmental challenges (reviewed in Dantzer et al, 2008) [1]. This communication is known to be bi-directional, with the CNS controlling immune function via neurotransmitters and hormones and the immune system modulating the CNS via cytokines and inflammatory mediators [2]. Some of the best characterized mechanisms on how the immune system influences brain function include direct actions of cytokines on neurons [3–5] as well as indirect cytokine actions on glial and perivascular cells [1, 6, 7]. Moreover, cytokines have been shown to induce the activation of the kynurenine pathway (KP) of tryptophan metabolism resulting in the formation of several neuroactive metabolites that modulate glutamatergic and cholinergic neurotransmission [8, 9]. Nevertheless, despite the vast information existing on the effects of inflammation and cytokines on neurotransmitter systems and behavior, defining their effects on higher order brain processes has proven challenging, as multiple mechanisms and possibly neurocircuitries are affected during an inflammatory reaction.

Resting state electro-encephalography (EEG) is a useful tool to understand brain circuitries and fast time scale information transfer in normal and pathological conditions [10, 11]. In humans, resting state EEG is a minimally invasive method to assess ongoing activity in the normal brain [12, 13], across psychiatric and neurological conditions [14–17], and following pharmacological manipulations [18–20]. Of translational value, rodent EEG measures correspond to, with a number of caveats, human EEG measures. For example, the power spectra at the surface of the cortex for both rodents and humans follow a similar frequency power profile [21]. Collectively, synchronized EEG activity reflects the sum of spatially distributed neuronal oscillations that serve ongoing sensory processing, affective modulation, and higher-order thought processes of the brain. These coupled oscillations arrange into networks at multiple spatial and temporal scales to coordinate, or bind, neural activity between regions [14]. Therefore, resting-state EEG recordings in multiple regions of the cortex may provide a powerful tool to understand the effects of inflammatory processes on higher order brain function and network processing.

Peripheral injection of subseptic doses of bacterial lipopolysaccharides (LPS) in rats and mice is an established model to elicit an inflammatory reaction and production of cytokines in the brain [22–28]. Depending on the dose and species, it produces a group of neurobehavioral symptoms known as sickness behavior, which persist from 2 to 24 hours [1, 28]. After cessation of the inflammatory reaction, these symptoms are followed by behavioral emotional and cognitive deficits. Cytokines are known to mediate the symptoms of sickness, while emotional and cognitive processing appears to be mediated by downstream mechanisms involving the KP [1, 29–32]. Thus, during an LPS challenge different inflammatory mediators interact with the CNS in a time-dependent manner raising the possibility of differential effects on synchronized cortical oscillatory activity during this process. Therefore, the objective of the present study was to use multi-channel resting state EEG recordings from the rat cortical surface to study the effects of the neuroinflammatory process elicited by LPS on EEG connectivity and power spectra. Results from this study confirm the assumption of a time-dependent effect of LPS on different components of cortical EEG during the progression and resolution of inflammation.

## Materials and methods

### Animals

Pregnant Wistar rats (Charles River Laboratories, Cambridge, MA) were received at our animal facility at gestational day 2 and allowed to deliver and nurse the offspring. Offspring were weaned at postnatal day (PND) 21 and one male offspring (n = 12) per dam was used in this study. Five days following weaning, animals were weighed and handled 3 times per week for three minutes per rat. Rats were implanted with surface electrodes at PND 54. Rats were maintained on a 12:12 L:D cycle (lights on at 07:00 AM) in plexiglass cages in groups of 2–3 per cage with food and water *ad libitum*. All animal procedures were approved by the Institutional Animal Care and Use Committee of the University of Maryland, Baltimore.

### Electrode implantation

**Fig 1:**
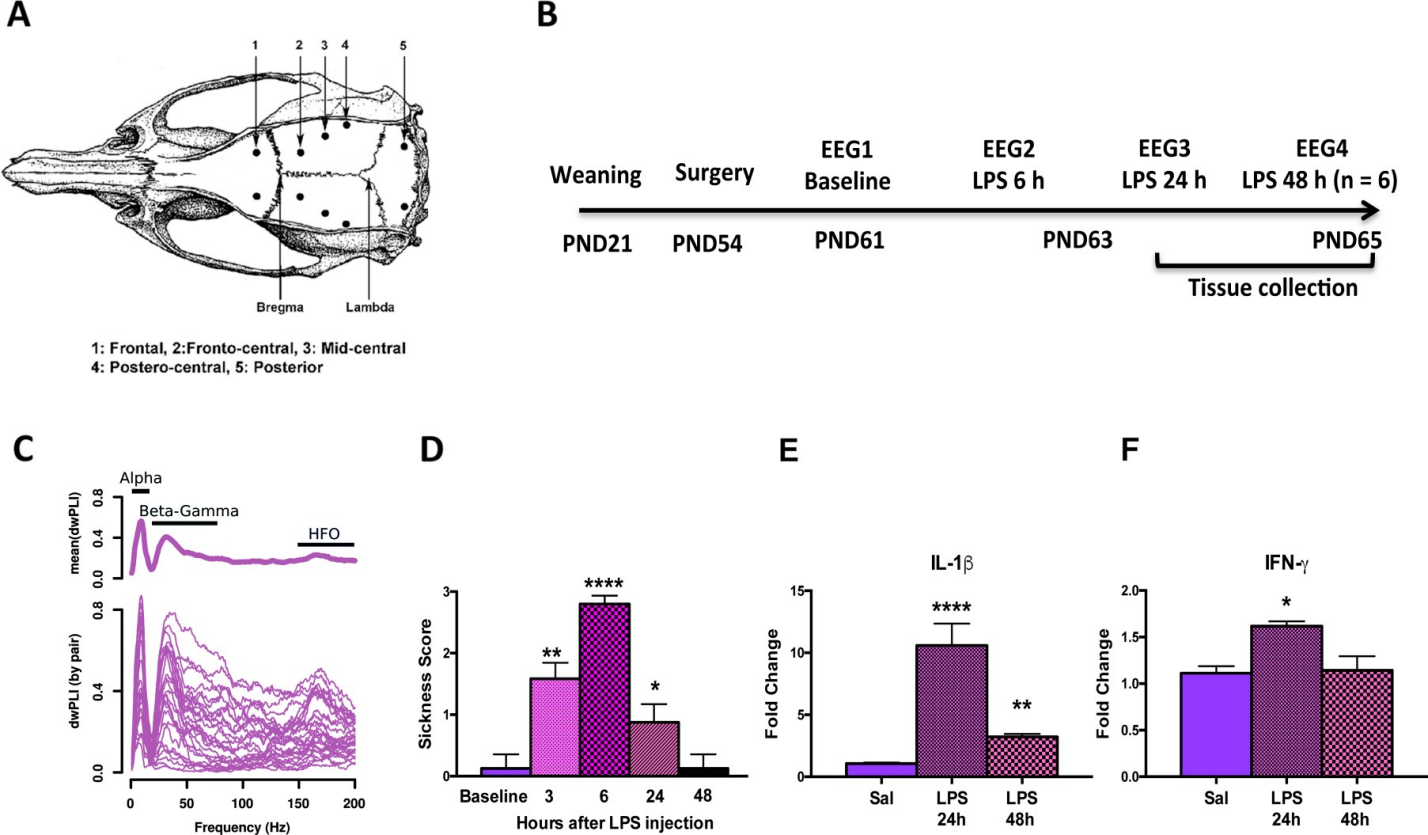
Study design and main experimental parameters. A: Position of the recording and reference electrodes in the rat skull modified from Paxinos and Watson. B: Time course of the experiments showing the recording sessions with respect to LPS administration. C: Resting state connectivity showing the frequency peaks defined by the debiased weighted phase lag index (dwPLI). Shown are the dwPLI pairs between all channels averaged over all baseline recordings at each frequency. The connectivity bands identified are denoted with top bars. D: Sickness behavior at baseline, 3, 6, 24 and 48 h after LPS administration. Kruskal-Wallis multiple comparison test with respect to baseline: * p = 0.02; ** p = 0.0011; **** *p* < 0.0001. E-F: Cytokine expression in the cerebral cortex and hippocampal formation of rats in saline treated or 24 and 48 h after LPS administration. Rats receiving saline injections (n = 6) were not used in recording experiments. LPS resulted in a strong response for interleukin-1 beta (IL-1β) (E) and a modest change in interferon gamma (IFN-γ) (F). * *p* = 0.05; **** *p* < 0.0001.

Rats were anesthetized with isoflurane and placed on a thermal pad to maintain temperature and monitored with a rectal thermometer. After ensuring a deep level of anesthesia, the top of the skull was shaved and one 2–3 cm incision was made along the midline of the scalp from just behind the line of the eyes to just in front of the ears. Ten burr holes not piercing the dura matter (5 on each side to the midline) were drilled into the skull at the following coordinates: frontal (2.0 mm lateral, 2.0 mm anterior to Bregma), fronto-central (2.0 mm lateral, 2.0 mm posterior to Bregma), mid-central (3.5 mm lateral, 4.0 mm posterior to Bregma), postero-central (5.0 mm lateral, 6.0 mm posterior to Bregma), posterior (3.0 mm lateral, 3.0 mm posterior to lambda) (Fig 1A). Stainless steel jeweler's screws (1.2 mm diameter) were used as electrodes with insulated wire leads soldered pre-surgically and implanted in each of the burr hole locations. The posterior electrodes served as ground and reference respectively. The free ends of the electrode leads were inserted using gold pins soldered pre-surgically into a 10 pin female-to-male connector. The entire assembly was secured to the skull using dental cement. The incision was closed around the head mount using wound clips (9 mm) and removed 7 days post-surgery. Following surgery, animals were placed into their home cages with a thermal pad and monitored until recovery. Monitoring continued daily including weight, overall appearance, fur condition, lack of grooming and signs of infection. All animals fully recovered without any signs of distress.

### LPS administration

Adult rats (PND 63) were injected intraperitoneally (i.p) with 2 mg/kg LPS (Sigma, St. Louis, MO, serotype 055:B5) between 09:00 and 10:00 AM and monitored at 3, 6, 24 and 48 h for temperature and sickness behavior using the 4 point scale as previously described [28, 33]. Briefly, rats were checked for lethargy (demonstrated by diminished locomotion after prompting and curled body posture), ptosis (drooping eyelids), and piloerection (ruffled, puffy fur) with each symptom equal to 1 point resulting in a scale of 0 to 3 with 0 = no symptom and 3 = all symptoms present. EEG recordings were acquired at baseline 48 h before injections, and at 6 and 24 h after LPS administration. Six rats were killed after the 24 h recording session and 6 underwent recordings at 48 h after LPS administration and killed after this recording session. At the completion of the recording sessions, the animals were brought in their home cages to a separated procedure room (one animal at the time) and killed by CO_2_ asphyxiation followed by decapitation and the brains removed and stored for cytokine determinations (Fig 1B).

### Recordings

Animals were allowed to acclimatize to the recording room for 1 h the day before the first EEG recording session. On the day of recording, animals were weighed and left undisturbed for 1 h before the head stage was connected to the head mount and recording began. Rats were individually housed and all recordings were obtained in the rat’s home cage. Recordings sessions included pairs or triplets, with cages side-by-side and with random allocation to recording position on each day. Immediately preceding the recordings, an observer rated the animals for sickness behavior and an experimenter remained in the room for the duration of the recording to ensure the integrity of the system. Data acquisition began immediately following application of the head stages to the animals. Resting state recordings were taken for 20 min. The EEG was obtained using a wireless telemetric 8-channel rodent electrophysiological recording system (ALA Scientific, Multichannel Systems–MCSW2100 system). Data were digitized at 2,000 Hz and EEG was continuously monitored for stable connections.

### EEG processing

EEG data were processed using the functions from EEGLAB [34]. For pre-processing, data were segmented into epochs of 500 milliseconds. Electrodes with sections of poor quality recordings were interpolated using a simple linear function using all available channels. Poor quality recording for a channel on an epoch by epoch basis was denoted as: any electrode with Spearman correlation coefficient with all other electrodes < 0.35 after applying a band pass filter between 1 and 210 Hz; a standard deviation of a channel's voltage > 350; or any point-to-point difference greater than 500 uV. Epochs with less than 6 usable channels were rejected. Further artifact rejection was applied to data with a standard deviation < 50, standard deviation >750, or a voltage exceeding ± 5000 uV. The data were then converted into 3 seconds epochs, before a final stringent artifact rejection threshold of ± 1000 μV. To determine if interpolation of the data altered the results, an independent sensitivity analysis without interpolation was also conducted and is presented as supplementary material. The results of both analyses are highly consistent, supporting an effect of LPS on power and connectivity.

### Power and connectivity

Power spectral density was calculated using the 'spectopo' function from EEGLAB, with a 1.5 seconds Hanning window, and a 50% overlap per epoch. Power derived from each epoch for each channel was then averaged across epochs. Band power was calculated as the average power with 6 commonly used frequency bands: delta (1–4 Hz), theta (4–8 Hz), alpha (8–12 Hz), beta (12–25 Hz), gamma (25–80 Hz), and gamma/high frequency oscillations (HFO; 80–200 Hz). Finally, band power was averaged across electrodes to provide a single number per rat per session for analysis. Connectivity was assessed using the debiased weighted phase lag index (dwPLI) using the available functions from FieldTrip [35]. The dwPLI is an extension of phase synchronization methods described by Nolte et al. (2004) and Stam et al. (2007) [36, 37], developed to minimize the effect of spurious connectivity arising from volume conduction and from the use of a common reference electrodes. The PLI takes the expectancy of the sign of the imaginary part of coherency, thereby reducing the dependence of the synchronization metric on the phase of the two signals. The dwPLI improves on the PLI by weighting the PLI by the magnitude of the imaginary part of coherency, and is defined as follows:

$${\hat{\Omega }}^{\omega }\equiv \frac{\sum_{j=1}^{Ν}\sum_{k\neq j}I\{{Χ}_{j}\}I\{{Χ}_{k}\}}{\sum_{j=1}^{Ν}\sum_{k\neq j}|I\{{Χ}_{j}\}I\{{Χ}_{k}\}|}$$

Where Xj and Xk are the complex valued cross-spectra of trials j and k, respectively [38]. Based on the shape of the connectivity profile as a function of frequency, three connectivity bands were defined for further analysis (Fig 1C). A peak around the alpha band as a central point with a decay on either side in the theta and beta bands as limits resulting in a connectivity frequency band of 5 to15 Hz. Similarly, a connectivity band in the beta-gamma range defined by a peak between 20 to 80 Hz, and a connectivity band with a peak in the HFO range between 150 to 200 Hz (Fig 1C). These connectivity bands were consistently identified in all experimental conditions. Connectivity was averaged within frequency bands across channels for representation in the figures. For analysis, connectivity was averaged over channel pairs within or across hemispheres.

### Phase-amplitude coupling

The connectivity analysis was supplemented with an analysis of phase-amplitude coupling using the modulation index from Tort et al. (2010) [39]. In brief, low frequency carrier signals were band pass filtered between 4 and 16 Hz, using 2 Hz step sizes and 4 Hz bandwidths with an additional 3 Hz carrier frequency included. The high frequency signals were filtered between 30 and 200 Hz using 5 Hz steps and 10 Hz bandwidths. The filtered signals were then Hilbert transformed, and the phase and amplitude of the low and high frequency signals were extracted. The phases were binned into 20 intervals and the mean amplitude of the high frequency signaled over each bin was calculated. Lastly, to obtain the modulation index, the mean amplitude was normalized by dividing the bin value by the sum over the bins (see Tort et al., 2010 for more details)[39].

### Real-time RT-PCR

Brains were dissected and the cerebral cortex and hippocampus from one hemisphere were pooled and processed for RNA extraction as described previously [40]. Five hundred ng of total RNA per sample were reverse transcribed into cDNA in a 20 μl reaction volume using an iScript cDNA Synthesis Kit (Bio-Rad, Hercules, CA, USA) according to manufacturer's instructions and then diluted 1:1 with ultrapure water. Real-time RT-PCR was conducted using the iQ SYBR Green Supermix (Bio-Rad) in a 25 μl reaction using the set of primers listed in S1 Table. Melting curves confirmed the generation of a single amplification product per gene (S1 Fig). Relative expression was determined using the 2^-ΔΔCt^ method [41].

### Statistical analysis

Data were prepared for analysis by importing the power and connectivity metrics obtained from Matlab into R (version 3.4.4; R Development Core Team: http://www.R-project.org). Power, connectivity, and phase-amplitude coupling were averaged into bands before being entered into statistical analysis. Hierarchical Bayesian repeated measure ANOVAs were employed to analyze resting state power and connectivity using the “rstan” package in R for Hamiltonian Monte Carlo sampling, a form of Markov chain Monte Carlo sampling. Kernel density estimates of the averaged and difference scores between sessions were plotted to assess suitability for analysis. All analyses were also rendered robust to outliers departing from normality through the use of a t-distribution to model the residuals. These t-distributions are wider tailed versions of the normal distribution where the degrees of freedom parameter (df) controls the width of the tails. The analysis is also able to explicitly take into the heterogeneity of variance by allowing for different variances to be fit to each condition. The measure possesses an excellent Type I error rate when used for multi-channel data as shown using permutation testing [20, 42, 43]. For the analysis of power and phase-amplitude coupling, treatment time (baseline, 6h, 24h) was treated as a within-subjects factor, while for connectivity, treatment and hemisphere (left, right) were treated as within-subjects factors. All models were run using 4 chains of 4,000 samples each. The first 2,000 samples of each chain were discarded as burn-in and adaptation. Convergence was monitored using the Gelman-Rubin statistic and the total number of effective samples per parameter of interest was > 2,000. Priors considered as minimally informative were scaled according to the data (2.5 * SD), and centered on a mean difference of 0 across sessions. To assess differences between groups, 95% highest density intervals (HDI) derived from each model's posterior were used [44, 45].

## Results

### Sickness behavior and cortico-hippocampal cytokine expression

Overt signs of sickness behavior were evident in all animals at 3 h after administration of LPS, with maximal signs at 6 h. Symptoms were beginning to resolve at 24 h and were completely absent by 48 h post LPS administration (Kruskal-Wallis test = 43.2, *p* < 0.0001) (Fig 1D). This response corresponds to a typical behavioral profile at this dose of LPS in rats [46], which is related to the inflammatory response elicited by i.p. LPS administration. Likewise, cytokine expression was dominated by a robust IL-1β response measured at 24 and 48 h post LPS administration and a modest IFN-γ response at 24 h (Fig 1E and 1F). No differences were observed on TNF-α expression at these time points.

### Spectral power: LPS induces a slowing of the resting-state EEG

**Fig 2:**
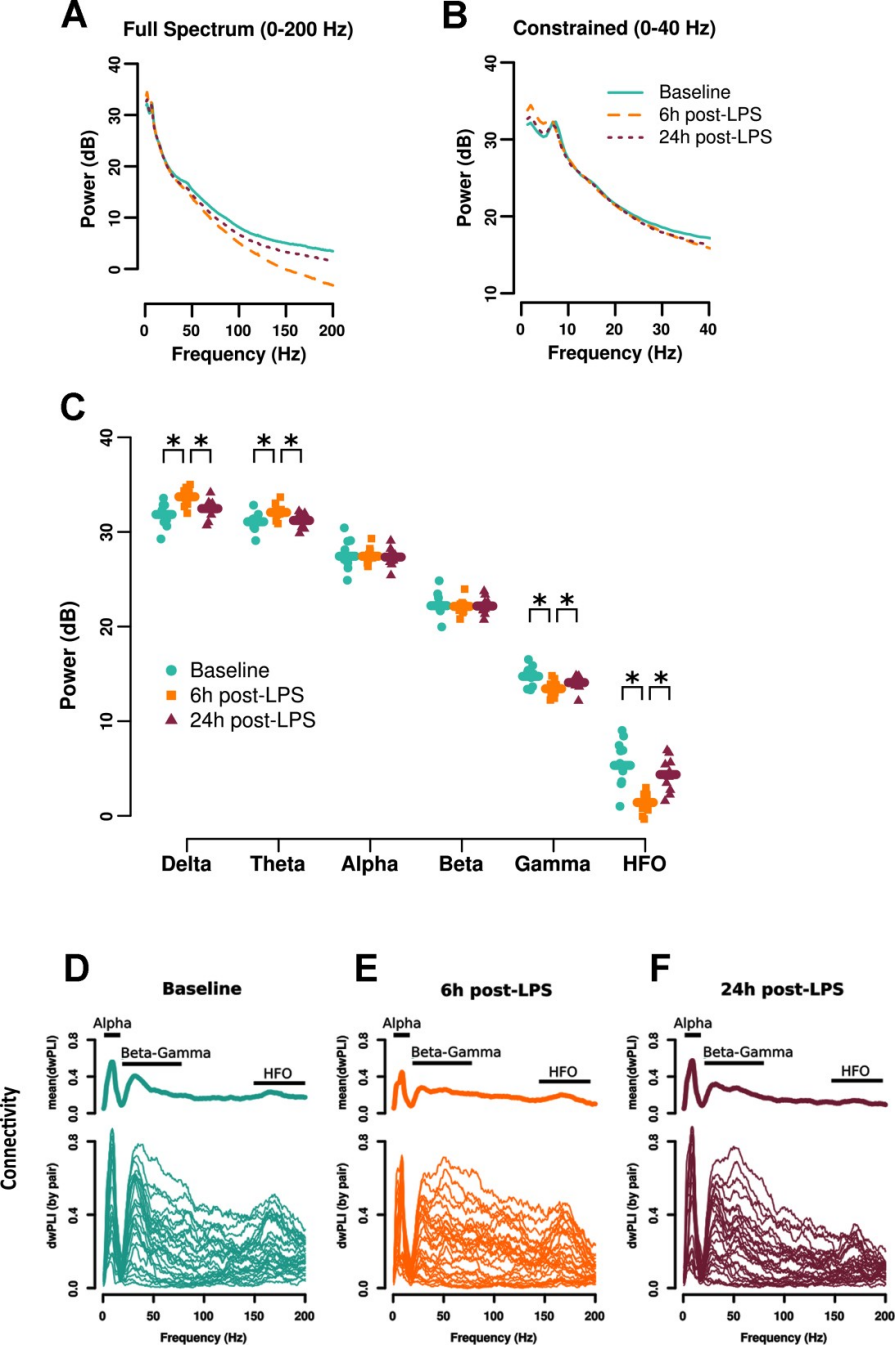
LPS: Spectral power and connectivity bands. Resting state power profile viewed across the full analyzed spectrum (1 to 200 Hz) (A) and constrained to 40 Hz (B) allowing better visualization of the lower frequencies. C: Power was averaged over all electrodes for each condition to yield a global measure of spectral power. Delta and theta power were increased 6 h after LPS administration (squares), while gamma power and high frequency oscillations (HFO) were reduced at this time point. Power in all affected frequency bands returned to baseline 24 h following LPS. Symbols (*) and lines indicate the 95% HDI contrast between any two conditions obtained from the posterior of the hierarchical Bayesian analysis excluded 0 (n = 11). D-F: Graphical visualization of the effect of LPS on resting state connectivity using the debiased weighted phase lag index (dwPLI). Each trace represents an electrode pair. Note the reductions in the alpha and beta-gamma connectivity bands 6 h after LPS and in the beta-gamma and HFO bands 24 h after LPS.

The power spectrum averaged over all electrodes at each time point following LPS administration is presented across all frequencies up to 200 Hz (Fig 2A) or constrained to 40 Hz (Fig 2B). The power follows the anticipated '1/f' power-frequency relationship with an alpha peak present between 7 and 9 Hz. The overall trace of the 6 h post-LPS power spectrum suggests a slowing of the EEG before returning to baseline 24 h post-LPS. Averaged power spectra from common defined bands including delta (1–4 Hz), theta (4–8 Hz), alpha (8–12 Hz), beta (12–25 Hz), gamma (25–80 Hz), and HFO (80–200 Hz) at each time point of study are shown in Fig 2C. Hierarchical Bayesian analysis confirmed the slowing of the EEG at 6 h post-LPS administration, with increases in the delta (6 h vs baseline contrast = 1.8 dB, 95% HDI = [1.0, 2.7]) and theta bands (6 h LPS vs baseline contrast = 1.0 dB, 95% HDI = [0.48, 1.5]) (Fig 2C). Both delta and theta power returned to baseline at 24 h post-LPS (delta 24 h vs baseline contrast = 0.53 dB, 95% HDI = [-0.38, 1.4]; theta 24 h vs baseline contrast = 0.15 dB, 95% HDI = [-0.42, 0.67]). Consistent with a slowing of EEG activity at 6 h after LPS, high frequency power was reduced in the gamma (6 h vs baseline contrast = -1.3 dB, 95% HDI = [-1.9, -0.62]) and HFO bands (6 h vs baseline contrast = -4.0 dB, 95% HDI = [-5.4, -2.5]). Both frequency bands returned to baseline at 24 h post-LPS administration (gamma 24 h vs baseline = -0.69, 95% HDI = [-1.4, -0.05]; HFO 24 h vs baseline = -1.0 dB [-2.7, 0.8]; Fig 2C). The same pattern was observed in the same animals that underwent recording at 48 h (S3 Fig). In contrast to the power changes in the low and high frequency bands, power in the alpha and beta bands were not affected by LPS administration at any time point (alpha 6 h vs baseline = -0.01 dB, 95% HDI = [-0.68, 0.57]; alpha 24 h vs baseline = -0.08 dB, 95% HDI = [-0.52, 0.29]; beta 6 h vs baseline = -0.07 dB, 95% HDI = [-0.50, 0.32]; beta 24 h vs baseline = -0.03, 95% HDI = [-0.51, 0.38]) (Fig 2C).

Supplemental sensitivity analysis using minimally processed data without interpolation (S3 Fig) produced similar results, demonstrating highly consistent effects of LPS on power with those presented above.

### Connectivity

#### LPS reduces connectivity

**Fig 3:**
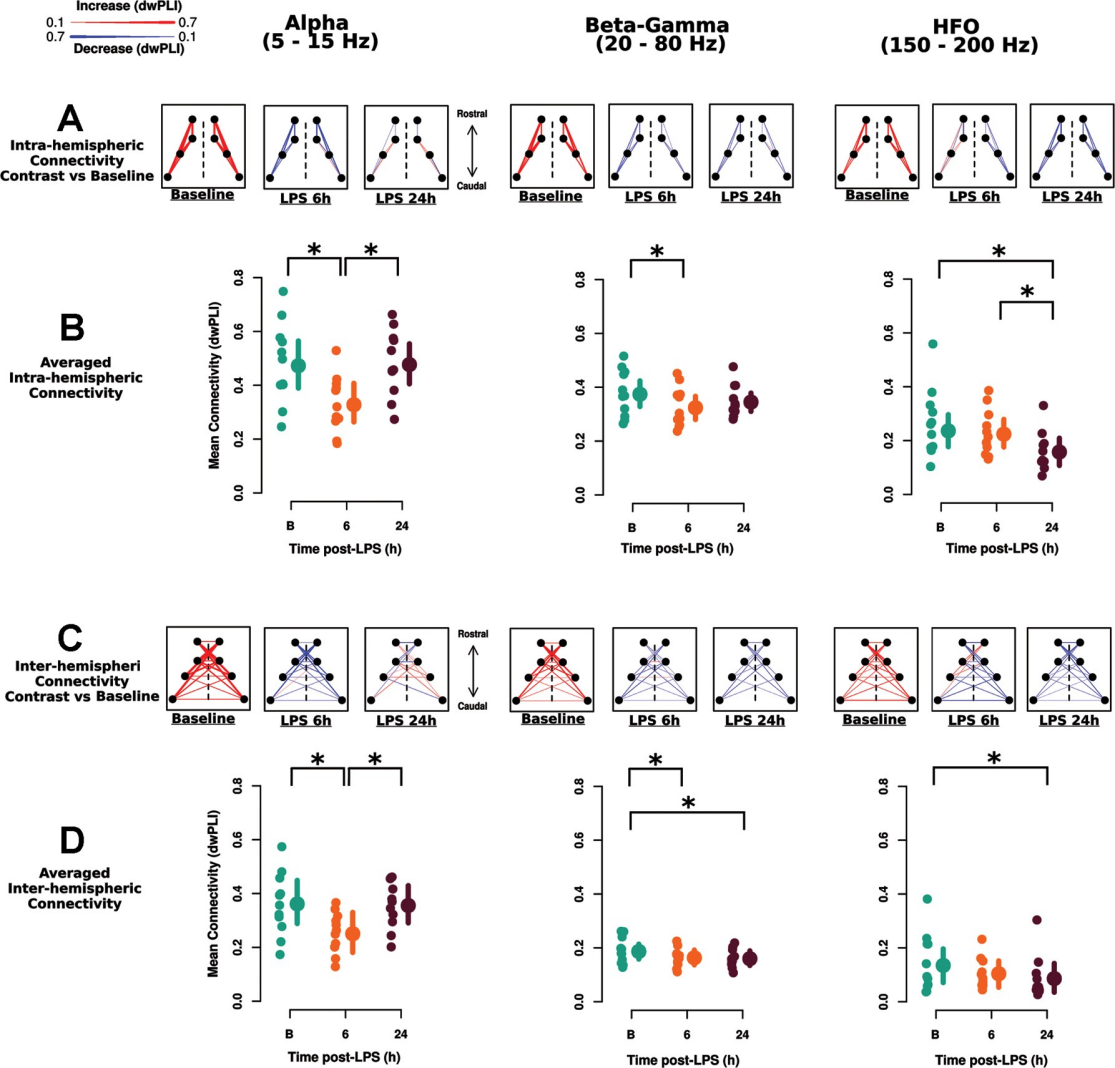
LPS reduces connectivity. Resting state connectivity reductions elicited by LPS. The debiased weighted phase lag index (dwPLI) was used as measure of connectivity between channels. Connectivity topography graphs for each of the 3 frequency bands analyzed (alpha, 5–15 Hz; beta-gamma 20–80 Hz and HFO 150–200 Hz) denoting the connection 'strength' between electrodes separated by intra- (A) and inter- (C) hemispheric connections. Baseline connectivity is denoted in red, with the strength of each connection represented by line width. Changes in connectivity between 6 or 24 h (contrast) with baseline are represented with red (increases) and blue (decreases) with the width of the line denoting the magnitude of change. Note that baseline effects are expected to be positive due to properties of the dwPLI. For analysis, connectivity was averaged over all channel pairs either within (B) or between (D) hemispheres to yield an average measure of inter- vs intra-hemispheric connectivity. No hemisphere by time interactions were detected indicating similar effects of LPS across hemispheres. Data and analyses are presented for both hemispheres for completeness. LPS reduced intra- and inter-hemispheric alpha connectivity at 6 h and returned to baseline at 24 h. Beta-gamma connectivity was reduced at 6 h (intra- and inter-hemispheric) and remained reduced 24 h (inter-hemispheric) after LPS. By contrast, HFO connectivity (intra- and inter-hemispheric) were reduced at 24 h after LPS administration.

Connectivity was analyzed using the dwPLI for intra- and inter-hemispheric channel pairs averaged within each of the 3 defined frequency bands (see methods). The overall effects of LPS between all electrode pairs are shown in Fig 2D–2F, with robust effects in the alpha (5–15 Hz) and beta-gamma (20–80 Hz) connectivity bands at 6 h and in the beta-gamma and HFO (150 to 200 Hz) bands at 24 h after LPS. Furthermore, strength-weighted connectivity between each pair of recording electrodes are presented in Fig 3A and 3C. Baseline along with 6 and 24 h deviations (baseline subtracted) for intra- (Fig 3A) and inter- (Fig 3C) hemispheric pairs connectivity are represented by blue (decrease) and red (increase) connecting lines. Averaged overall intra- (Fig 3B) and inter- (Fig 3D) hemispheric connectivity are shown for individual animals. Both intra- and inter-hemispheric alpha connectivity was reduced 6 h following LPS (intra- and inter-hemispheric 6 h vs baseline contrast = -0.13, 95% HDI = [-0.18, -0.08]). Alpha connectivity returned to baseline 24 h post-LPS (intra- and inter-hemispheric 24 h vs baseline contrast = 0.00, 95% HDI = [-0.04, 0.05]). Likewise, beta-gamma connectivity was also reduced at 6 h following LPS across both hemispheres (6 h vs baseline = -0.04, 95% HDI = [-0.07, -0.01]) (Fig 3A–3D) and remained suppressed at 24 h post-LPS (24 h vs baseline = -0.03, 95% HDI = [-0.05, -0.004]). HFO connectivity was also reduced across both intra- and inter- hemispheric pairs (24 h vs baseline contrast = -0.07, 95% HDI = [-0.10, -0.04]), but there was no reduction in HFO at 6 h post-LPS (6 h vs baseline contrast = -0.02 [-0.05, 0.005]). The same pattern of results was seen in the sub-sample of 6 rodents who underwent a 48 h recording post-LPS with a return to baseline at 48 hours post-LPS (S4 Fig). No interactions between hemisphere and time of LPS administration were found in any frequency band. Supplemental sensitivity analyses of minimally processed data show highly consistent effects of LPS on connectivity to that presented above (S5 Fig).

#### Phase-amplitude coupling: LPS increases theta-gamma coupling

**Fig 4:**
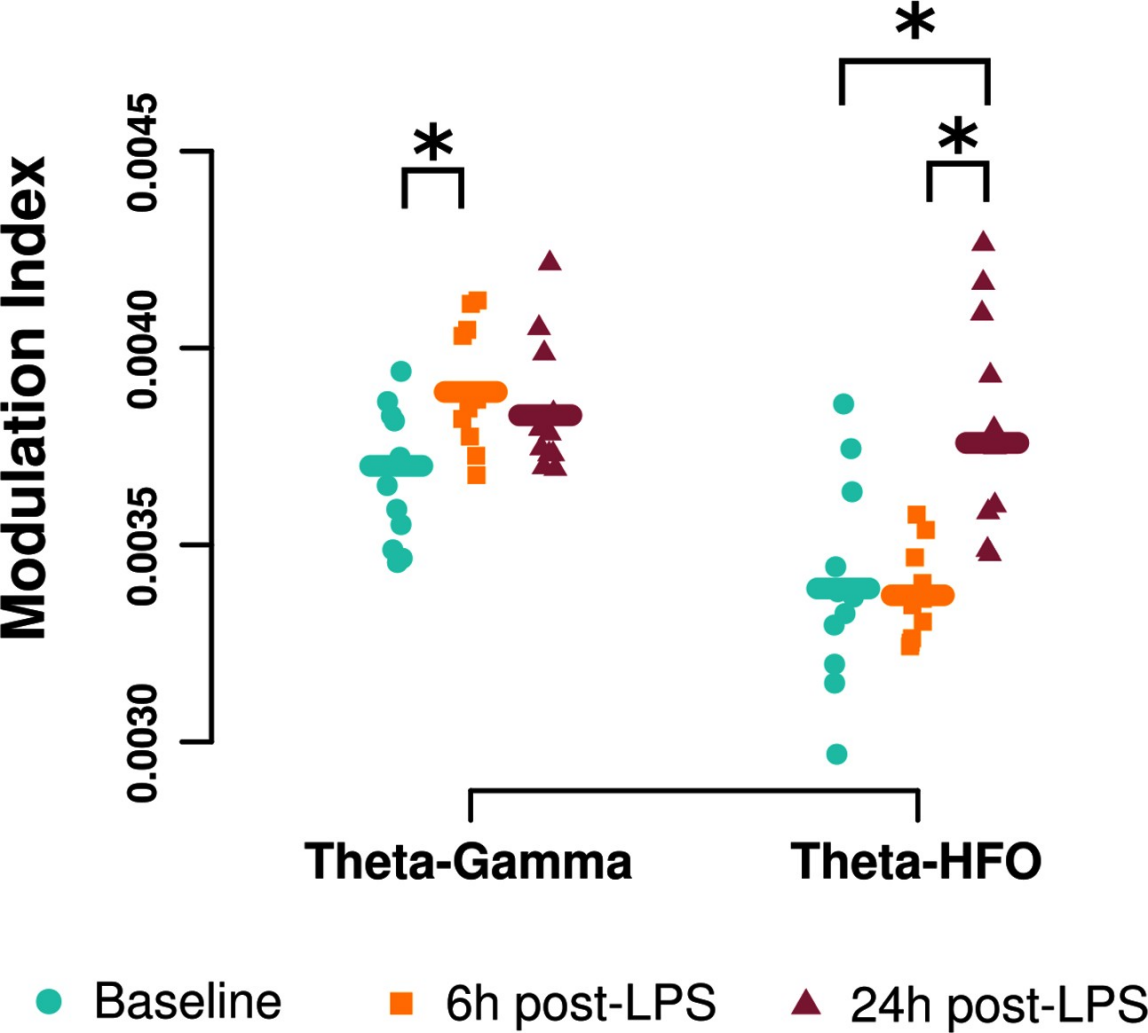
LPS increases phase-amplitude coupling. Resting state phase-amplitude coupling increased following LPS. The modulation index (MI) was used as a measure of phase-amplitude coupling between theta (3–8 Hz) phase and gamma power (30–100 Hz), and theta phase and HFO (150–200 Hz) power. The MI was averaged over all electrodes for each condition to yield a global measure of the MI. The theta-gamma MI was increased 6 h after LPS administration (squares). Interestingly, the theta-HFO MI was increased only at the 24 h time point relative to both baseline and 6h post-LPS. Symbols (*) and lines indicate the 95% HDI contrast between any two conditions obtained from the posterior of the hierarchical Bayesian analysis excluded 0 (n = 11).

Phase-amplitude coupling was analyzed using the modulation index obtained from two cross-frequency interactions: theta-gamma (3–8 Hz carrier and 30–100 Hz amplitude) and theta-HFO (3–8 Hz carrier and 150–200 Hz amplitude), as is common in the literature [39]. The theta-gamma modulation index was increased 6 h following LPS administration (6 h vs baseline contrast = 0.00019, 95% HDI = [0.00003, 0.00034]) (Fig 4). While it appeared that theta-gamma coupling remained elevated at 24 h post-LPS, the 95% HDI did not reveal a difference (24 h vs baseline contrast = 0.00013, 95% HDI = [-0.00002, 0.00027]). Interestingly, theta-HFO coupling was increased at 24 h post-LPS (24 h vs baseline contrast = 0.00037, 95% HDI = [0.0001, 0.0006]), but not at 6 h (6 h vs baseline contrast = -0.00002, 95% HDI = [-0.00022, 0.00015]) (Fig 4).

## Discussion

The present studies revealed a time-dependent effect of a single dose of LPS (2 mg/kg) on the rat resting state EEG. LPS caused an overall slowing of the EEG 6 h following LPS administration, increasing slow wave power (delta and theta bands) and reducing high frequency power (gamma and HFO bands), with a return to baseline 24 h after injection. LPS reduced intra- and inter-hemispheric connectivity across all frequency bands analyzed in a time- and frequency-dependent manner showing that the neuroinflammatory processes elicited by peripheral LPS disrupts both bottom-up and top-down communication across the cortex during the peak and resolution of inflammation. The present results also indicate that while power and connectivity are related measures of EEG activity, these processes are independently affected by LPS and in a time dependent manner.

### LPS-induced reductions in resting state EEG functional connectivity are temporally sustained in the high frequency range

Despite an increasing interest in the study of functional coupling between brain regions, the literature on the effects of inflammatory states on functional connectivity appear limited to a recent fMRI study [47]. Using EEG, inferring connectivity has imposed significant challenges as EEG-related measures of connectivity are strongly confounded by volume conduction and the use of a common reference electrode [38]. Recent connectivity methods such as the dwPLI used in the current study greatly reduce the influence of these confounders [38]. To our knowledge, these methods have not yet been applied to rodent surface recordings that are conceptually similar to human EEG recordings and may possess significant translational value. Using this approach, 3 connectivity bands were identified and clearly defined across both experiments, which closely relate to the bands identified with local field potential recordings in the dentate gyrus and CA3 regions of the hippocampus [48]. The most prominent connectivity band was a well-defined peak in the 5 to 15 Hz range. Interestingly, a similar peak in connectivity is seen around the alpha band in human resting state EEG studies using the dwPLI [20, 36]. Two other connectivity bands were identified from the dwPLI spectra, one covering the beta-gamma frequencies from 20 to 80 Hz and another higher frequency band covering 150 to 200 Hz (Fig 1C).

Administration of LPS substantially reduced alpha connectivity 6 h following LPS administration returning to baseline 24 h later. It is important to note that the generation of alpha oscillations (reflected by the alpha power value) was not affected by LPS administration. This indicates that power and dwPLI connectivity are independent components and are affected differentially by inflammation. Alpha power modulation is observed during cognitive processes that require top-down control and inhibition of irrelevant sensory information [49, 50]. Thus, LPS-induced reductions in alpha connectivity specifically (and not power) may reflect an inability to coordinate the inhibition of irrelevant information. Alternatively, the overall reduced external sensory processing elicited during the acute phase of the inflammatory reaction [1] may render the functional significance of alpha coordination less necessary. These effects on alpha connectivity were robust 6 h after LPS and coincident with the full manifestation of the behavioral symptoms of sickness including lethargy, curled posture, piloerection and ptosis and are logically associated with this behavioral state. Alpha connectivity returned to baseline 24 h after LPS at the time that these behavioral symptoms subsided, further suggesting a relationship between reduced sensory processing and alpha connectivity during the LPS challenge.

Remarkable effects of LPS on EEG resting state connectivity were observed in the high frequency range covering the beta-gamma (20 to 80 Hz) and HFO (150 to 200 Hz) peaks. Reductions in beta-gamma connectivity were observed at 6 h after LPS, at the time that power was also reduced in this band. Furthermore, reductions in both beta-gamma and HFO connectivity were present 24 h after LPS when power returned to baseline levels further supporting the notion that these two measures reflect closely related but yet distinct phenomena. As mentioned before, at this time-point the behavioral symptoms of sickness have subsided and the animals recover most of the psychomotor functions affected during the acute phase. However, extensive studies in the literature, including work from our group, have shown that motivation and emotional processing remain affected during this period of recovery from sickness [1, 28, 30, 32, 46]. It is possible then that persistent reduction in connectivity in these bands 24 h after LPS are directly associated with reduced motivational and cognitive processing elicited by LPS challenge. Under the framework of several computational theories [51–53] gamma and other high frequency oscillations are currently believed to drive feed-forward activations that most prominently, but not exclusively, originate from sensory cortices and travel “up” the cortical hierarchy. Beta-gamma connectivity reductions tentatively represent a combined impairment of feedforward and feedback communication, with the beta band likely serving as a fast feedback prediction signal and the gamma band serving a predominantly efferent/feedforward prediction error signal. Similarly, reduced HFO connectivity likely indicates a failure of the effectiveness of feedforward communication in the cortex, despite the ability to generate HFO at baseline strengths 24 h post-LPS, highlighting that reduced HFO connectivity is not merely a function of a reduction in signal strength. It would be relevant to test to what extent the transfer of information rather than the generation of the oscillations, is responsible for the reduced motivational component during recovery from LPS challenge. Furthermore, future studies could examine the effects of neurotransmitter modulators co-administered at the time of the peak of the inflammatory reaction to assess the neurotransmitter systems affecting connectivity during LPS challenge.

### LPS increases theta-gamma and theta-HFO coupling

Cross frequency coupling, and in particular theta-gamma coupling, has been proposed to represent a form of neural communication and computation that emerges during high order brain functions [54, 55]. In brief, the amplitude of high frequency oscillations can be modulated by the phase of lower ‘carrier’ frequencies during encoding of complex behavioral processes. Phase-amplitude coupling is often studied in the context of memory operations, targeting hippocampal-fronto cortical communications [39, 56–58]. To our knowledge, theta-gamma coupling has not yet been studied in resting state EEG in animals undergoing an inflammatory challenge. Our data indicated time-dependent increases of theta-gamma and theta-HFO coupling elicited by LPS occurring in a temporally-dependent manner. This is, theta-gamma coupling was increased at 6 h and theta-HFO at 24 h after LPS. While speculative at this point, these effects are consistent with the concept of and increased effort of neural networks to maintain cognitive processing. For example, Tamura et al. (2017) [58] recently elegantly demonstrated increases in theta-gamma coupling in a genetic mouse model of cognitive dysfunction during a spatial working memory task. The increase in coupling was suggested to reflect a compensatory mechanism to maintain behavioral performance, a notion further supported by optogenetic and behavioral manipulations showing increased theta-gamma coupling with task difficulty [58]. This “compensatory” effect of theta-gamma coupling is consistent with recent findings of increased resting-state cross-frequency coupling in people with Alzheimer’s disease [59]. This was also suggested to reflect an increase in the neuronal resources required to maintain the resting brain state and potentially, an attenuated complexity of the neuronal network [59]. Further studies may assess the possibility of a compensatory role for increased cross-frequency coupling elicited by LPS through assessment of explicit spatial working memory tests.

### LPS induced expected changes in EEG spectral power

LPS is known to cause an increase in low frequency power, in particular delta power, associated with sleep alterations [60–63]. Our study showed increases in delta and theta power 6 h after LPS, which coincides with the peak of sickness behavior. Moreover, our study further reports on decreased power on the gamma and HFO bands at 6 h post LPS. Although we did not measure sleep, visual scoring of sickness symptoms confirmed increased lethargy and curled posture typical of sleep in rats at 6 h post LPS. Moreover, the time course of EEG disruption over the session was analyzed under the assumptions that the animals would be less likely to be asleep immediately after attaching the head stage on them. This analysis (S6 Fig) shows that the effects reported in this study are present within the first 10 minutes of recording, suggesting that the effects are unlikely driven primarily due to sleep. Notably, power for all frequency bands was restored 24 h after LPS supporting the notion that power and connectivity are affected by different mechanisms elicited by LPS. However, this assumption was not evaluated in the present experiments and will be a matter of future studies.

### Potential neuroimmune interactions during LPS relevant to the effects on cortical EEG

Peripheral administration of LPS in rats causes a robust and widespread expression of the cytokine IL-1β across the entire CNS, which varies in its regional distribution and level of expression in a temporal manner [22–25, 64–66]. Both the hippocampus and cortex respond with widespread expression of IL-1β produced mainly by microglial cells [23, 25]. This is accompanied by the expression of additional cytokines including TNF-α, IL-6 and IFN-γ and inflammatory mediators such as nitric oxide (NO) [67, 68]. The modulatory actions of these cytokines on neuronal electrical activity have been documented by several studies [69–75]. Moreover, direct effects of IL-1β on GABAergic and glutamatergic neurotransmission have been described by a number of studies [69, 72, 74]. Furthermore these cytokines, as well as LPS, increase kynurenine metabolism in the brain resulting in activation of the KP [76, 77] producing several neuroactive metabolites that modulate glutamatergic neurotransmission [8, 78]. Of note, kynurenic acid, a metabolite of the KP pathway, which acts as an endogenous NMDA antagonist [8], is increased by LPS challenge [79, 80]. Thus, the effects of LPS on EEG spectra may primarily be the result of interference with glutamatergic and GABAergic neurotransmission in a temporally dependent manner. This may be reflected by the increases in low frequency power at earlier time-points followed by changes in higher frequency bands at later time-points. Moreover, we have recently shown that kynurenic acid producing astrocytes are concentrated in white matter tracts including the corpus callosum [40], therefore effects of LPS on connectivity were expected and confirmed. Of interest, future studies could examine the effects of GABA and glutamate modulators co-administered at different times of the inflammatory reaction to assess the neurotransmitter systems affected during LPS challenge.

## Conclusions

The present studies using multi-electrode array recordings of resting state EEG in rats identified several connectivity bands that were significantly impacted by the neuroinflammatory process triggered by peripheral administration of LPS. These effects were time-dependent and coincided with different behavioral states associated with the emergence and resolution of the symptoms of sickness. These studies reveal specific effects of inflammation on brain EEG functional connectivity, thereby contributing to our understanding of the impact of neuroinflammation on mechanisms linking the immune response with higher order brain functions.

## Supporting information

S1 Table Primer set used for cytokines in RT-PCR. Sequences used in real-time RT-PCR determinations. IL-1β: interleukin-1 beta; IFN-γ: interferon gamma; TNF-α: tumor necrosis factor alpha; 18S: ribosomal 18s rRNA; TFRC: transferrin receptor.
(TIF)

S1 Fig Melting curves for the set of primers used in RT-PCR determinations. A: Melting peaks for a single PCR run under the same amplification conditions for the control genes 18s and transferrin receptor (TFRC) and the target genes interleukin-1 beta (IL-1β), tumor necrosis factor alpha (TNF-α) and interferon gamma (IFN-γ). B-D: Isolated melting peaks for IL-1β, TNF-α and IFN-γ respectively. NTC: non-template control.
(TIF)

S2 Fig Resting state power for the subset of animals that underwent an additional 48 h recording. Power was averaged over all electrodes for each condition to yield a global measure of spectral power. The findings in this subset (n = 6) strongly mimic the findings from the full cohort, including: delta and theta power increases 6 h after LPS administration (squares), and gamma and high frequency oscillation (HFO) power reductions at this time point. A complete return to baseline can be seen at 48 h following LPS for all frequencies. Symbols (*) and lines indicate that the 95% HDI contrast between any two conditions obtained from the posterior of the hierarchical Bayesian analysis excluded 0.
(TIF)

S3 Fig Resting state power of minimally processed data. Resting state power profile of data processed without interpolation equivalent to Fig 2. A: Full analyzed spectrum (1 to 200 Hz). B: Spectrum constrained to 40 Hz allowing better visualization of the lower frequencies. C: Power was averaged over all electrodes for each condition to yield a global measure of spectral power. Delta and theta power were increased 6 h after LPS administration (squares), while gamma power and high frequency oscillations (HFO) were reduced at this time point. Power in all affected frequency bands returned to baseline 24 h following LPS (same as Fig 2).
(TIF)

S4 Fig Resting state connectivity for the subset of animals that underwent an additional 48 h recording. Averaged intra- (A) and inter- (B) hemispheric connectivity in the 3 frequency bands identified with the dwPLI for the subset of animals that completed 48 h recordings (n = 6). As in the whole sample, alpha connectivity was reduced at 6 h and returned to baseline at 24 h and maintained at 48 h. Persistent beta/gamma reductions were seen at 24 h, as well as reductions in intra- and inter-hemispheric HFO connectivity at 24 h. HFO connectivity impairments were mostly restored at 48 h.
(TIF)

S5 Fig Connectivity of minimally processed data. Averaged connectivity measures of data without interpolation equivalent to that presented in Fig 3. No hemisphere by time interactions were detected indicating similar effects of LPS across hemispheres. LPS reduced intra- and inter-hemispheric alpha connectivity at 6 h and returned to baseline at 24 h. Beta-gamma inter-hemispheric connectivity was reduced at 6 h and remained reduced 24 h after LPS. By contrast, HFO connectivity (intra- and inter-hemispheric) were reduced at 24 h after LPS administration.
(TIF)

S6 Fig Time course of the EEG power for the full 20 min recording for each analyzed frequency band. The data is presented as a 5-point moving average (i.e., each data point is five 3 s epochs). Changes in low frequency power (delta, theta, alpha, and beta) were most evident 6 h following LPS administration during the first 10 minutes of recording. By comparison, LPS-induced reductions in high frequency power bands (gamma and HFO) were persistent across the whole recording period. At 24 h following LPS administration, the power time-course in each frequency band was consistent with baseline, with the exception of the high frequency power bands. High frequency bands showed gradual reductions over the 20 min recording session at 24 h following LPS.
(TIF)
