# Supplementary figures and images for: Time and frequency dependent changes in resting state EEG functional connectivity following lipopolysaccharide challenge in rats
Source: PLoS One. 2018 Nov 12;13(11):e0206985. doi: 10.1371/journal.pone.0206985 (PMC6231634; doi:10.1371/journal.pone.0206985)

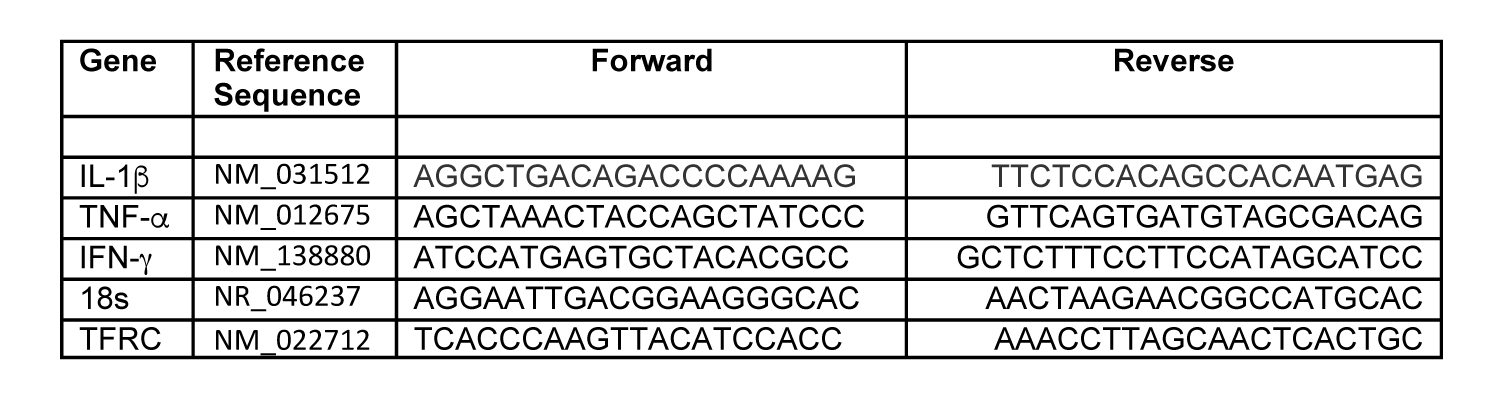

Supplement: S1 Table — Sequences used in real-time RT-PCR determinations. IL-1β: interleukin-1 beta; IFN-γ: interferon gamma; TNF-α: tumor necrosis factor alpha; 18S: ribosomal 18s rRNA; TFRC: transferrin receptor. (TIF) [file pone.0206985.s001.tif]

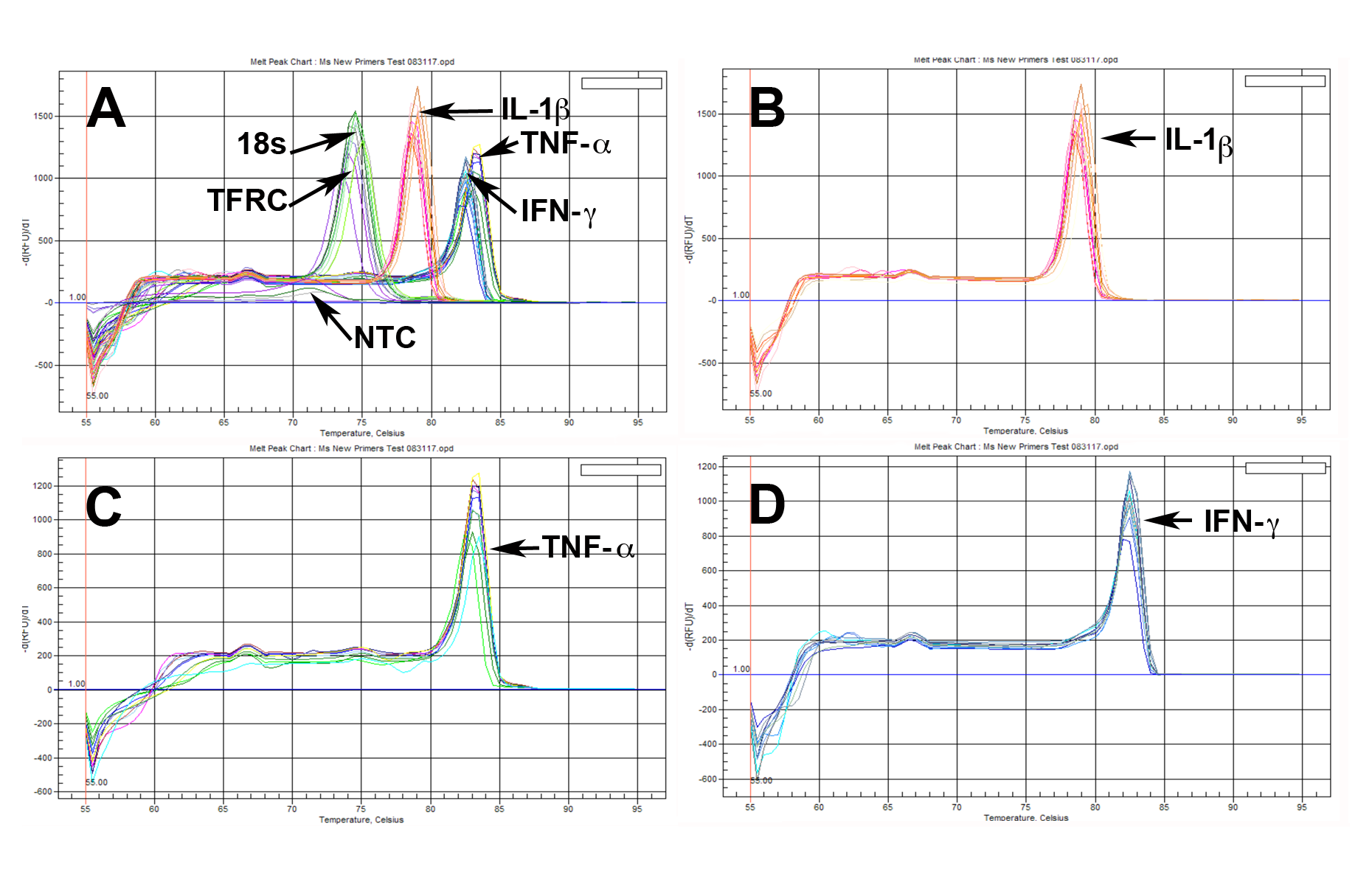

Supplement: S1 Fig — A: Melting peaks for a single PCR run under the same amplification conditions for the control genes 18s and transferrin receptor (TFRC) and the target genes interleukin-1 beta (IL-1β), tumor necrosis factor alpha (TNF-α) and interferon gamma (IFN-γ). B-D: Isolated melting peaks for IL-1β, TNF-α and IFN-γ respectively. NTC: non-template control. (TIF) [file pone.0206985.s002.tif]

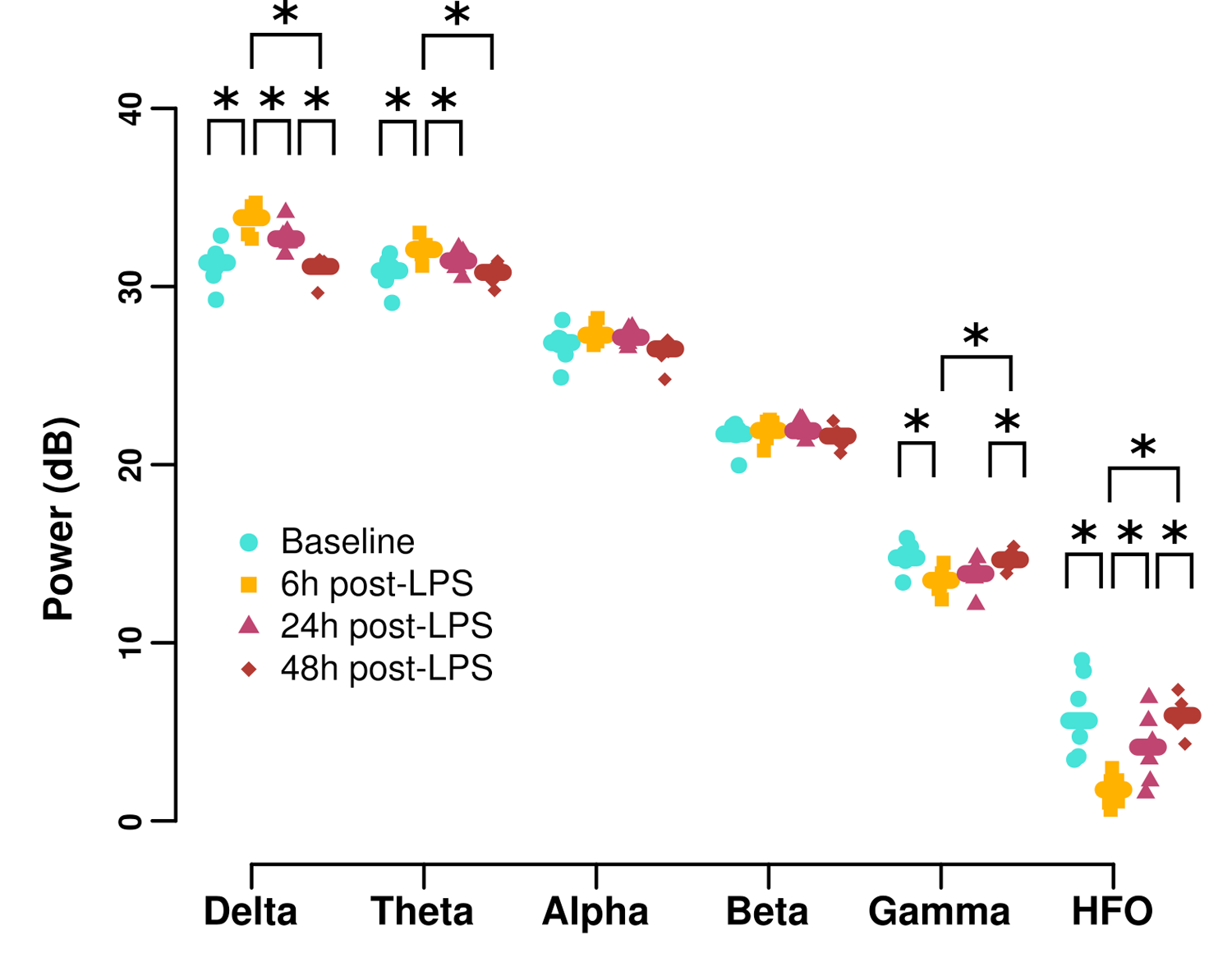

Supplement: S2 Fig — Power was averaged over all electrodes for each condition to yield a global measure of spectral power. The findings in this subset (n = 6) strongly mimic the findings from the full cohort, including: delta and theta power increases 6 h after LPS administration (squares), and gamma and high frequency oscillation (HFO) power reductions at this time point. A complete return to baseline can be seen at 48 h following LPS for all frequencies. Symbols (*) and lines indicate that the 95% HDI contrast between any two conditions obtained from the posterior of the hierarchical Bayesian analysis excluded 0. (TIF) [file pone.0206985.s003.tif]

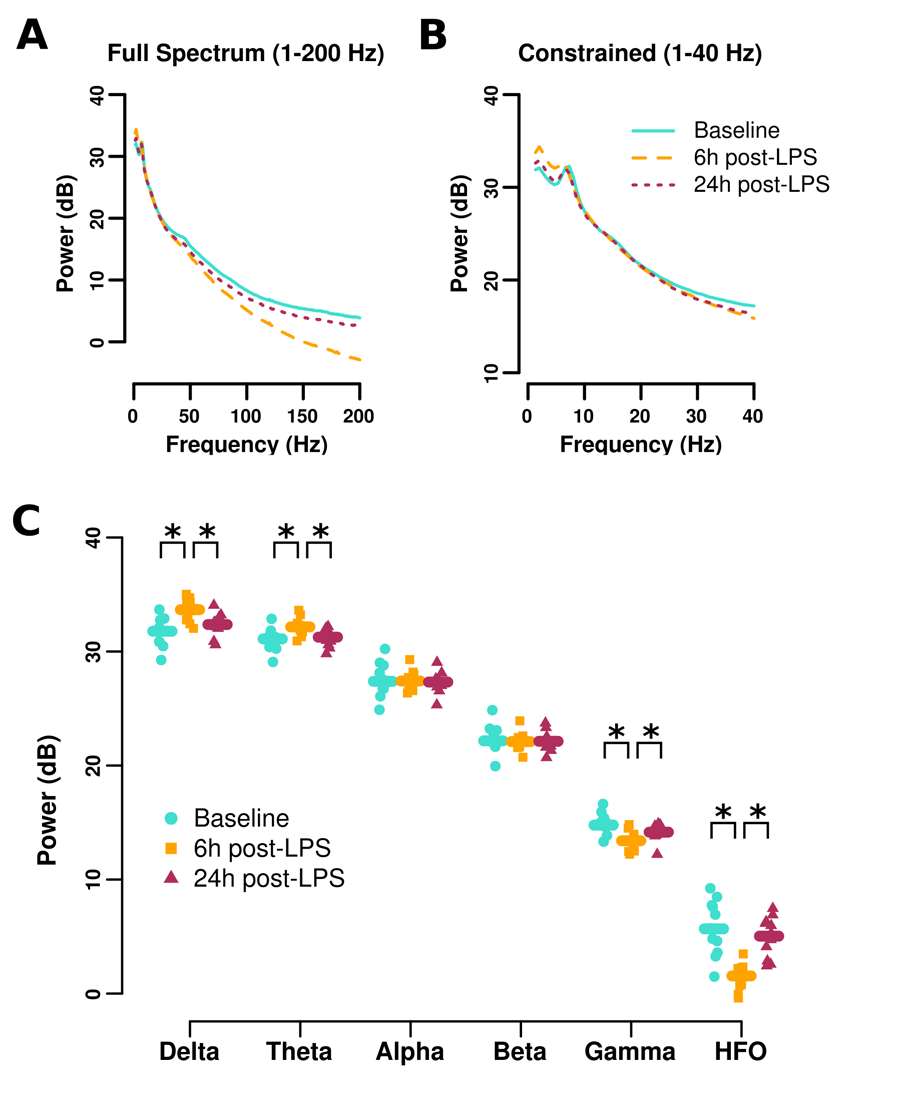

Supplement: S3 Fig — Resting state power profile of data processed without interpolation equivalent to Fig 2. A: Full analyzed spectrum (1 to 200 Hz). B: Spectrum constrained to 40 Hz allowing better visualization of the lower frequencies. C: Power was averaged over all electrodes for each condition to yield a global measure of spectral power. Delta and theta power were increased 6 h after LPS administration (squares), while gamma power and high frequency oscillations (HFO) were reduced at this time point. Power in all affected frequency bands returned to baseline 24 h following LPS (same as Fig 2). (TIF) [file pone.0206985.s004.tif]

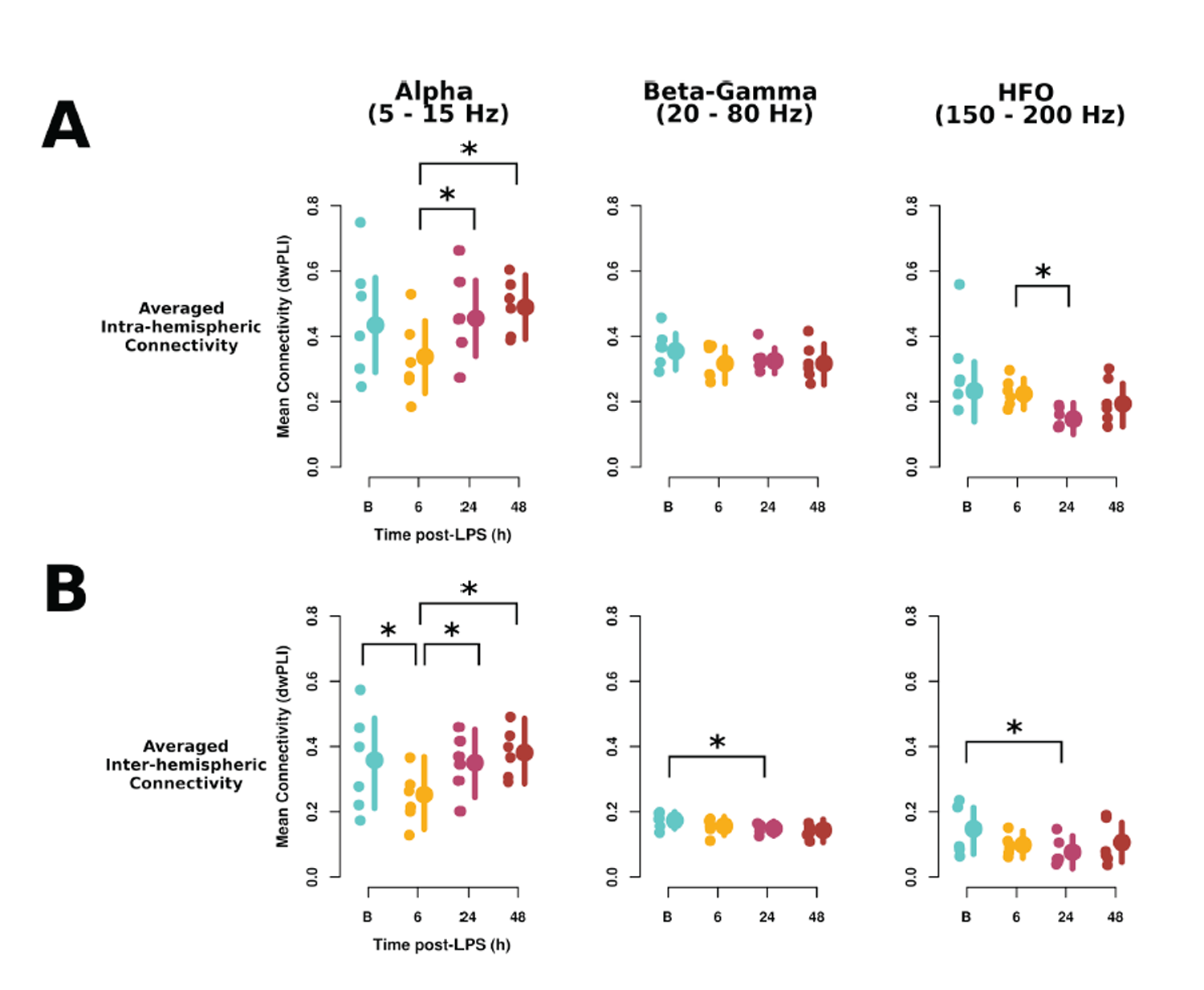

Supplement: S4 Fig — Averaged intra- (A) and inter- (B) hemispheric connectivity in the 3 frequency bands identified with the dwPLI for the subset of animals that completed 48 h recordings (n = 6). As in the whole sample, alpha connectivity was reduced at 6 h and returned to baseline at 24 h and maintained at 48 h. Persistent beta/gamma reductions were seen at 24 h, as well as reductions in intra- and inter-hemispheric HFO connectivity at 24 h. HFO connectivity impairments were mostly restored at 48 h. (TIF) [file pone.0206985.s005.tif]

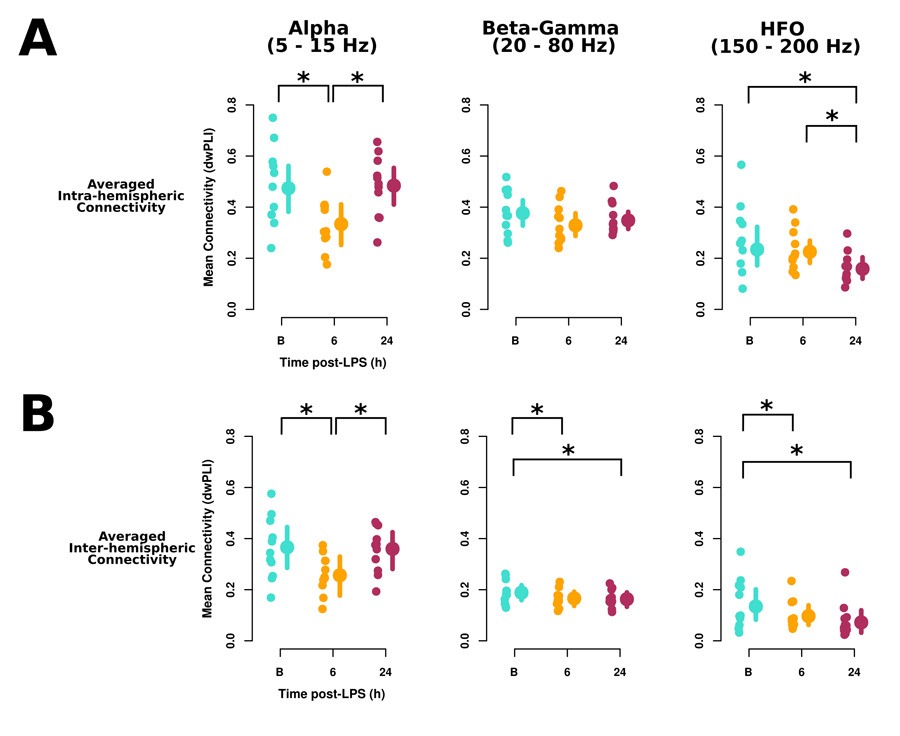

Supplement: S5 Fig — Averaged connectivity measures of data without interpolation equivalent to that presented in Fig 3. No hemisphere by time interactions were detected indicating similar effects of LPS across hemispheres. LPS reduced intra- and inter-hemispheric alpha connectivity at 6 h and returned to baseline at 24 h. Beta-gamma inter-hemispheric connectivity was reduced at 6 h and remained reduced 24 h after LPS. By contrast, HFO connectivity (intra- and inter-hemispheric) were reduced at 24 h after LPS administration. (TIF) [file pone.0206985.s006.tif]

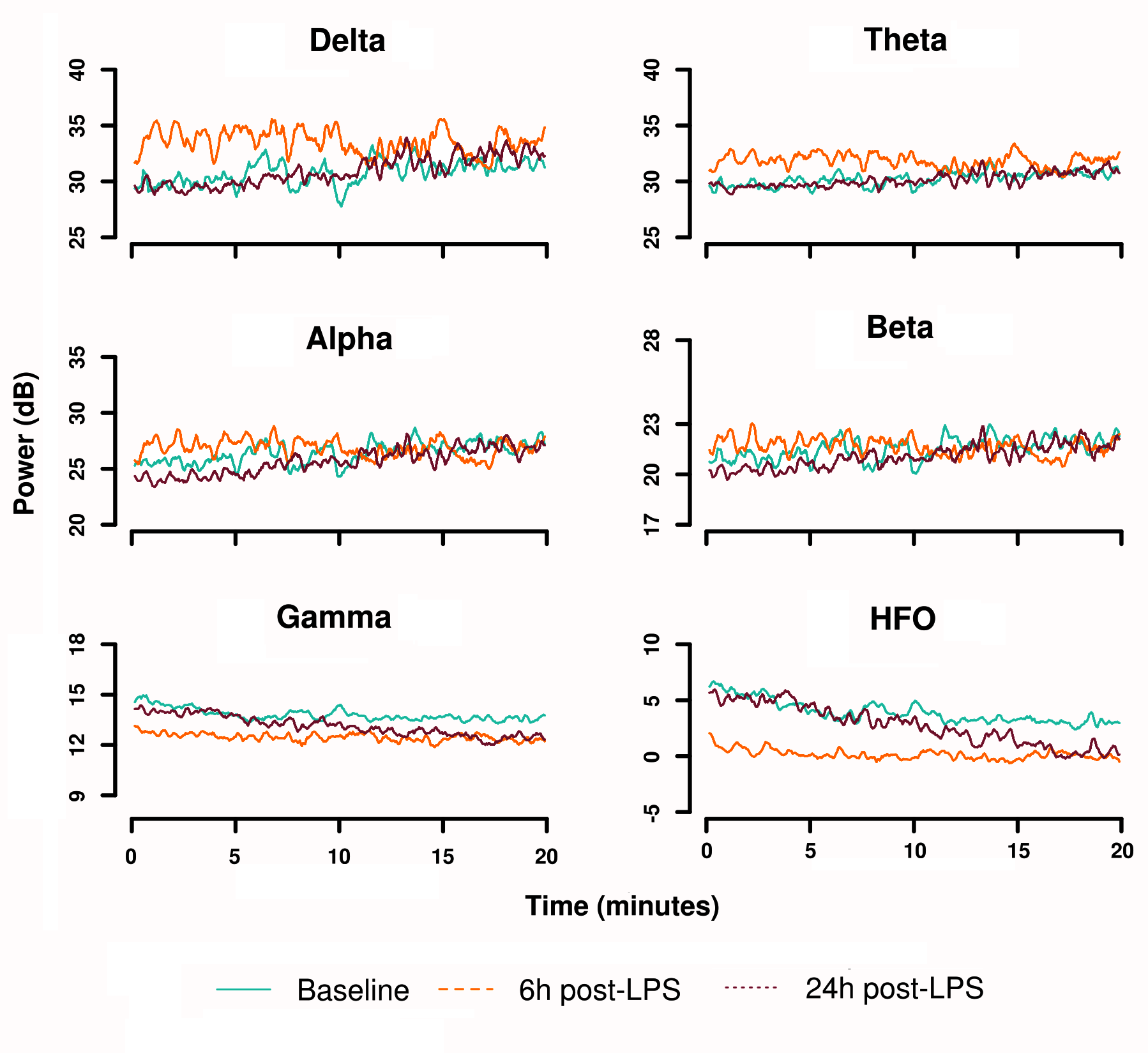

Supplement: S6 Fig — The data is presented as a 5-point moving average (i.e., each data point is five 3 s epochs). Changes in low frequency power (delta, theta, alpha, and beta) were most evident 6 h following LPS administration during the first 10 minutes of recording. By comparison, LPS-induced reductions in high frequency power bands (gamma and HFO) were persistent across the whole recording period. At 24 h following LPS administration, the power time-course in each frequency band was consistent with baseline, with the exception of the high frequency power bands. High frequency bands showed gradual reductions over the 20 min recording session at 24 h following LPS. (TIF) [file pone.0206985.s007.tif]
